# Thiocoraline alters neuroendocrine phenotype and activates the Notch pathway in MTC-TT cell line

**DOI:** 10.1002/cam4.118

**Published:** 2013-09-17

**Authors:** Sara Tesfazghi, Jacob Eide, Ajitha Dammalapati, Colin Korlesky, Thomas P Wyche, Tim S Bugni, Herbert Chen, Renata Jaskula-Sztul

**Affiliations:** 1 University of Wisconsin Medical SchoolMadison, Wisconsin; 2 University of Minnesota Medical SchoolMinneapolis, Minnesota; 3 Department of Surgery, University of Wisconsin Medical SchoolMadison, Wisconsin; 4 Department of Biomedical Engineering, University of WisconsinMadison, Wisconsin; 5 Department of Pharmacy, University of WisconsinMadison, Wisconsin

**Keywords:** Cell cycle, medullary thyroid cancer, neuroendocrine cancer, Notch signaling, thiocoraline

## Abstract

Medullary thyroid cancer (MTC) is an aggressive neuroendocrine tumor (NET). Previous research has shown that activation of Notch signaling has a tumor suppressor role in NETs. The potential therapeutic effect of thiocoraline on the activation of the Notch pathway in an MTC cell line (TT) was investigated. Thiocoraline was isolated from a marine bacterium *Verrucosispora* sp. MTT assay (3-[4, 5-dimethylthiazole-2-yl]-2, 5-diphenyltetrazolium bromide) was used to determine the IC_50_ value and to measure cell proliferation. Western blot revealed the expression of Notch isoforms, NET, and cell cycle markers. Cell cycle progression was validated by flow cytometry. The mRNA expression of Notch isoforms and downstream targets were measured using real-time PCR. The IC_50_ value for thiocoraline treatment in TT cells was determined to be 7.6 nmol/L. Thiocoraline treatment decreased cell proliferation in a dose- and time-dependent manner. The mechanism of growth inhibition was found to be cell cycle arrest in G1 phase. Thiocoraline activated the Notch pathway as demonstrated by the dose-dependent increase in mRNA and protein expression of Notch isoforms. Furthermore, treatment with thiocoraline resulted in changes in the expression of downstream targets of the Notch pathway (HES1, HES2, HES6, HEY1, and HEY2) and reduced expression of NET markers, CgA, and ASCL1. Thiocoraline is a potent Notch pathway activator and an inhibitor of MTC-TT cell proliferation at low nanomolar concentrations. These results provide exciting evidence for the use of thiocoraline as a potential treatment for intractable MTC.

Thiocoraline is a potent Notch pathway activator and an inhibitor of medullary thyroid cancer cell line (MTC-TT) cell proliferation at low nanomolar concentrations. These results provide evidence for the use of thiocoraline as a potential treatment for intractable MTC.

## Introduction

Medullary thyroid cancer (MTC) is a malignancy of the parafollicular C cells of the thyroid gland 1,2. Most MTCs are sporadic (80%), while ∼20% of cases are inherited as a germline mutation in the *re*arranged during *t*ransfection (*ret)* proto-oncogene 1–4. MTCs can present as an aggressive malignancy with metastases to the liver, lungs, bone, and mediastinum 2,5,6. Hormones secreted by C cells include chromogranin A (CgA), synaptophysin (SYP), and calcitonin and are found to be elevated in patients with MTC 1,7,8. In addition, neuroendocrine tumor (NET) markers like the transcription factor achaete–scute complex-like 1 (ASCL1) are highly expressed in MTC cells 8,9. Previous research has shown ASCL1 to be critical for C cell development and to be important in MTC tumor growth 4,10.

Many patients (50–80%) present with metastatic disease at the time of diagnosis 11. Surgery is the primary treatment for MTC, but the majority of patients undergoing resection will develop recurrent disease 1,3. Novel therapies that target signaling pathways regulating cell proliferation are therefore needed for the effective management of MTC.

Thiocoraline, a thiodepsipeptide bisintercalator, has been shown to be cytotoxic in lung, breast, colon, renal, and melanoma cancer cells, and studies have shown that it induces G1 cell cycle arrest 12–14. Additionally, previous research has shown that thiocoraline has an antiproliferative effect on human colon adenocarcinoma cell lines by arresting cells in G1 phase as well as decreasing the rate of S phase progression toward G2/M 13,15. For this study, thiocoraline was isolated and purified after production by a marine bacterium (*Verrucosispora* sp.), cultivated from the sponge *Chondrilla caribensis f. caribensis* 12. The downstream target pathways of thiocoraline are not well described.

We have shown that induction of Notch1 is associated with inhibition of MTC tumor growth in vivo 16. In addition, it is known that activated Notch1 causes a reduction in ASCL1 expression 4. Accordingly, the effect of thiocoraline as a potential Notch pathway activator in MTC was investigated. The Notch signaling pathway is comprised of four transmembrane proteins, Notch1–4 that are proteolytically cleaved upon ligand binding 8,17,18. Following cleavage, the Notch intracellular domain (NICD) is released from the receptor and translocates to the nucleus where it binds and forms a complex with centromere binding factor 1 (CBF-1) 19,20. This complex causes the induction of downstream genes including the hairy enhancer of split (HES) and HES-related (HEY) families of transcription regulators 19. HES1 in particular is significant because it binds to the promoter of ASCL1 and downregulates transcription 21.

In this study, we describe the effects of thiocoraline on human MTC cell line (TT). On the basis of the antiproliferative effect of thiocoraline on other cancer cell lines, we hypothesize that thiocoraline treatment may have therapeutic potential in MTC-TT. As our previous work has shown that the Notch pathway is minimally active in MTC-TT cells, we wanted to examine the potential of thiocoraline to induce the expression levels of Notch isoforms (Notch1 and Notch2), which would lead to a subsequent reduction in NET markers ASCL1, CgA, calcitonin, and SYP.

This study is the first to investigate the anti-tumor effects of thiocoraline on MTC cells and the results provide compelling evidence for the use of thiocoraline as a potential treatment for intractable MTC.

## Materials and Methods

### Cell culture

Human MTC-TT was provided by Dr. Barry D. Nelkin (John Hopkins University, Baltimore, MD). The integrity of TT cells as distinct cell line were confirmed by short tandem repeat (STR) profile testing and the genotype of the TT cells is available in the American Type Culture Collection (ATCC) STR database. TT cells were maintained in RPMI 1640 medium (Life Technologies, Grand Island, NY) supplemented with 16% fetal bovine serum (Sigma, St. Louis, MO), 100 IU/mL penicillin (Life Technologies), and 100 μg/mL streptomycin (Life Technologies) in a humidified atmosphere of 5% CO_2_ in air at 37°C 4,22.

### Thiocoraline

**Figure 1:**
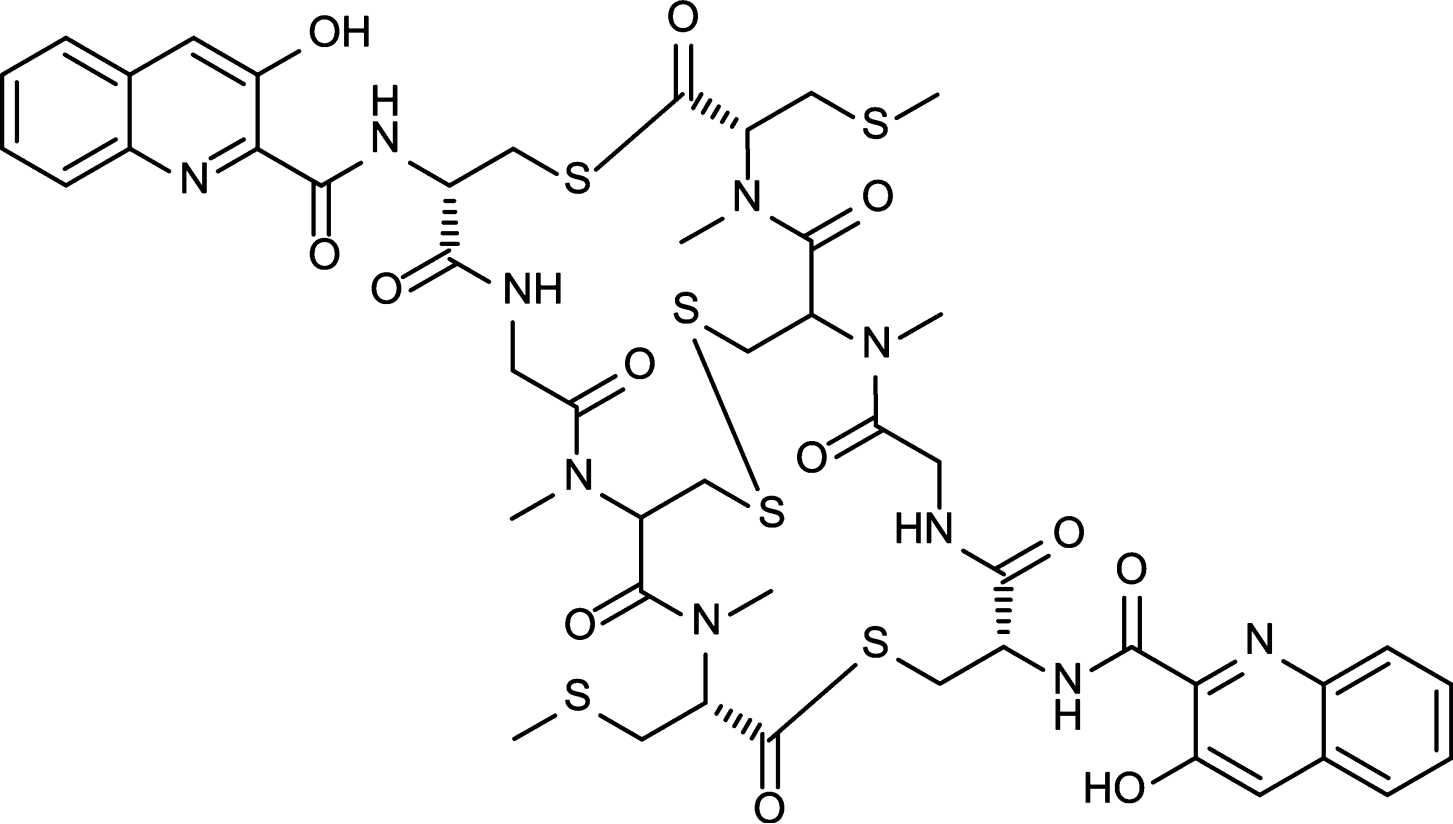
The chemical structure of thiocoraline. Thiocoraline, isolated from a marine *Verrucosispora* sp., is a bisintercalator. The 3-OH-quinaldic system, which has been proposed to stabilize the complex with DNA, provides thiocoraline with a unique mechanism of action and sequence specificity over other bisintercalators, which contain a quinoxaline ring system 12. While bisintercalators containing the quinoxaline ring result in DNA damage and inhibition of topoisomerase II, thiocoraline inhibits DNA elongation via its interaction with DNA polymerase α.

*Chondrilla caribensis f. caribensis* sponge specimens were collected in the Florida Keys on 10 February 2010 as described by Wyche et al. 12. Thiocoraline was isolated and purified from the marine bacterium *Verrucosispora* sp. as previously described 12. Thiocoraline was dissolved in dimethyl sulfoxide (DMSO) and diluted in standard media to achieve desired concentrations (Fig. 1).

### Cell proliferation assay and IC_50_ determination

Cell proliferation was measured via 3-(4, 5-dimethylthiazol-2yl)-2, 5 diphenyltetrazolium bromide (MTT) assay as previously described 4,23. In brief, cells were plated in quadruplicates in 24-well plates under standard conditions. The following day cells were treated with thiocoraline (0–10 nmol/L) and incubated for up to 8 days. Control-treated cells (0 nmol/L) received DMSO at 0.005% final concentration. Cell proliferation was assessed after 2, 4, 6, and 8 days. Following 2 days of thiocoraline treatment, the dose effect curve was plotted to determine the IC_50_ value using CompuSyn 1.0 Software (ComboSyn Inc., Paramus, NJ). The MTT assay was performed by replacing the standard media with 250 μL of serum-free RMPI 1640 containing 0.5 mg/mL MTT and incubated for 3.5 h at 37°C. After incubation, 750 μL of DMSO was added per well and absorbance at 540 nmol/L was measured via a spectrophotometer (μQuant; Bio-Tek Instruments, Winooski, VT). To assess a decrease in the cell number, TT cells were stably transfected with lentivirus expressing red fluorescent protein (RFP) (Gen Target, Inc., San Diego, CA). After a 48-h treatment with DMSO or 5 and 10 nmol/L thiocoraline, TT cells were washed with phosphate buffered saline (PBS) and RFP-positive cells were counted under an inverted fluorescent microscope (Nikon Eclipse Ti, Melville, NY).

### Flow cytometry

To analyze the cell cycle progression of TT cells, the DNA content was quantified via flow cytometry. TT cells were treated for 2 days with thiocoraline concentrations of 0–10 nmol/L. After treatment, cells were washed with cold 1× PBS pH 7.2 (Life Technologies) and harvested with trypsin (Life Technologies). Cells were centrifuged at 1200 rpm at 4°C and washed twice with cold 1× PBS before being fixed with cold 70% ethanol and kept at −20°C before staining. Prior to staining, cells were again washed twice with cold 1× PBS with centrifugation after each wash. The pellet was subsequently suspended in a propidium iodide (PI) staining solution containing 20 mg/mL RNAse-A (Sigma) and 330 μg/mL propidium iodide dissolved in 1× PBS. Cells were stained in the dark overnight at 4°C. Samples were filtered prior to analysis. Fluorescence-activated cell sorting (FACS) analysis was performed on a flow cytometer at 488 nmol/L (FACSCalibur flow cytometer; BD Biosciences, San Jose, CA), and results were analyzed with ModFit LT 3.2 software (Verity, Topsham, ME).

### Western blot analysis

Following 2-day thiocoraline treatment (0–10 nmol/L) protein extracts were harvested and quantified as previously described 4,22. Denatured cellular extracts (30–40 μg) were resolved on 7.5% or 10% SDS-PAGE (sodium dodecyl sulfate polyacrylamide gel electrophoresis; Life Technologies), transferred onto nitrocellulose membranes (Bio-Rad Laboratories, Hercules, CA), blocked in milk solution 16, and incubated overnight in the appropriate primary antibody. The following primary antibodies were used at the following concentrations: anti-NOTCH1 (1:2000); anti-NOTCH2 (1:1000); anti-MASH1 (mammalian ASH1) to detect ASCL1 (1:2000; Pharmingen/BD, Franklin Lakes, NJ); anti-CgA (1:1000; Zymed Laboratories/Life Technologies); anti-p21 (1:2000); anti-p27 (1:2000); anti-cyclin B1 (1:1000), anti-cyclin D1 (1:1000), anti-HEY2 (1:1000; Abcam, Cambridge, MA) and anti-glyceraldehyde-3 phosphate (GAPDH) (1:10,000; Trevigen, Gaithersburg, MD). Membranes were washed prior to secondary antibody incubation 16. The following secondary antibodies at the indicated dilutions were used: goat anti-rabbit (Notch1 1:4000, Notch2 1:4000, Cyclin D1 1:2000, p27 1:6000, CgA 1:4000, GAPDH 1:3000); goat anti-mouse (Cyclin B1 1:3000, p21 1:6000, ASCL1 1:5000). Following secondary antibody incubation, proteins were visualized as previously described 4,16.

### Quantitative real-time polymerase chain reaction

Following 2-day thiocoraline treatment, RNA was isolated using RNeasy Mini-kit (Qiagen, Valencia, CA) and reverse transcribed with the iScript cDNA synthesis kit (Bio-Rad). Quantitative real-time polymerase chain reaction (PCR) was performed by the iCycler IQ detection system (Bio-Rad). A 25 μL volume reaction containing 2 μL cDNA sample (200 ng/μL), 200 nmol/L forward and reverse primers, and 12.5 μL SYBR Green Supermix (Bio-Rad) was used. The following PCR forward and reverse primer pairs were used: Notch1 (5′-GTCAACGCCGTAGATGACCT-3′ and 5′-TTGTTAGCCCCGTTCTTCAG-3′), Notch2 (5′-TGTGACATAGCAGCCTCCAG-3′ and 5′-CAGGGGGCACTGACAGTAAT-3′), HES1 (5′-TTGGAGGCTTCCAGGTGGTA-3′ and 5′-GGCCCCGTTGGGAATG-3′), HES2 (5′-CTC ATT TCG GAC CTC GGT T-3′ and 5′-TTC GAG CAG TTG GAG TTC T-3′), HES6 (5′-AGCTCCTGAACCATCTGCTC-3′ and 5′-GACTCAGTTCAGCCTCAGGG-3′), and s27 (5′-TCTTTAGCCATGCACAAACG-3′ and 5′-TTTCAGTGCTGCTTCCTCCT-3′), HEY1 (5′-CGAGGTGGAGAAGGAGAGTG-3′ and 5′-CTGGGTACCAGCCTTCTCAG-3′) as a loading control. The RT-PCR reactions were performed in duplicate under previously described conditions 16. Results were normalized to s27 mRNA levels and expression was plotted as average ± standard error of the mean (SEM).

### Statistical analysis

Flow cytometry analysis was performed using a one-way analysis of variance (ANOVA). Analysis of MTT growth curves was performed using a one-way ANOVA and the Kruskal–Wallis rank sum test. A value of *P* ≤ 0.05 was considered statistically significant.

## Results

### Thiocoraline inhibits MTC-TT cell proliferation in vitro

**Figure 2:**
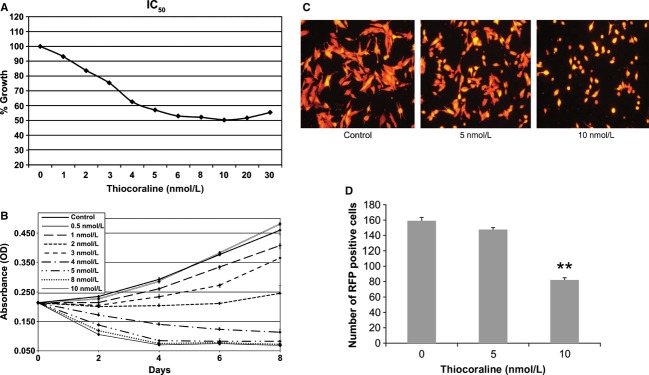
Thiocoraline inhibits TT cell proliferation in vitro. MTT assay was used to determine the IC_50_ value after 48 h of thiocoraline-treated TT cells (A). Cell viability was determined in a time and dose-dependent manner by MTT assay; 2-day treatments with concentrations greater than 0.5 nmol/L significantly decreased cell growth (*P* < 0.05). A highly significant (*P* < 0.01) decrease in proliferation was found for all treatments greater than 0.5 nmol/L after 4- and 6-day treatments, as well as a highly significant (*P* < 0.01) decrease for treatments greater than 1.0 nmol/L after 8 days. Experiments were done in quadruplicate and data are plotted as mean ± SEM (B). TT cells were stably transfected with lentivirus expressing red fluorescent protein (RFP) and counted under fluorescent microscope after 48 h of DMSO or thiocoraline (5 and 10 nmol/L) exposure (C). The number of TT cells in culture decreased 10% (not statistically significant) and 50% (**P < 0.01) when exposed to 5 and 10 nmol/L of thiocoraline, respectively (D).

Our first objective was to determine the effects of thiocoraline treatment on cell survival. A cell proliferation study determined that the IC_50_ value was 7.6 nmol/L (Fig. 2A), therefore doses between 0 and 10 nmol/L were used for cell treatment. A dose- and time-dependent decrease in cell proliferation was observed with increasing thiocoraline treatment (Fig. 2B). It was determined that there was a significant (*P* < 0.05) decrease in cell growth for concentrations greater than 0.5 nmol/L following 2-day treatment. A highly significant (*P* < 0.01) decrease in proliferation was found for all doses greater than 0.5 nmol/L after 4- and 6-day treatments, as well as a highly significant (*P* < 0.01) decrease for treatments greater than 1.0 nmol/L after 8 days. These results suggested that thiocoraline was a potent inhibitor of TT cell viability at low nanomolar concentrations. Additionally, Figure 2C demonstrates that thiocoraline reduces cell number with treatment as evidenced by measurement of RFP expression via fluorescent microscopy. As shown in Figure 2D, treatment with 10 nmol/L of thiocoraline decreased 50% (*P* < 0.01) TT cells in culture.

### Thiocoraline induces cell cycle arrest in MTC-TT cells

**Figure 3:**
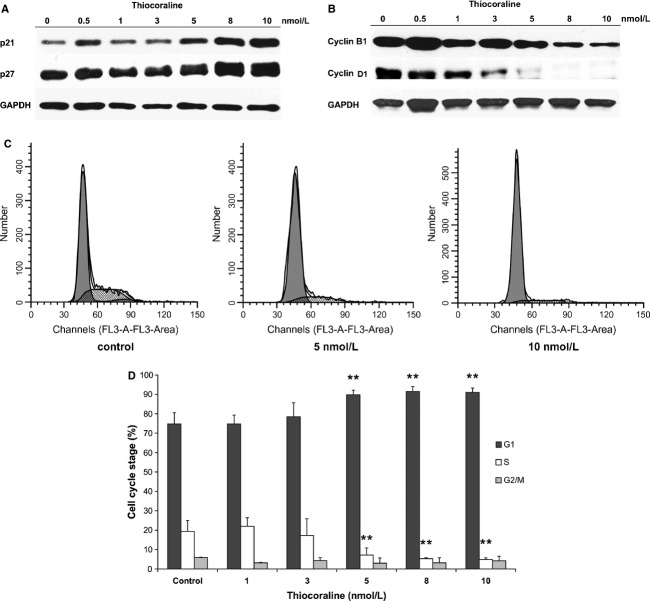
Thiocoraline suppresses TT cell proliferation through cell cycle arrest. Thiocoraline dose-dependent changes in the expression of cell cycle regulatory proteins (p21, p27 [A] and cyclin B1, cyclin D1 [B]) monitored by Western blots suggest cell cycle arrest as a mechanism of TT cell growth inhibition. GAPDH (glyceraldehyde-3-phosphate dehydrogenase) levels indicate equal loading. Flow cytometry analysis revealed that thiocoraline induces G1 phase arrest (C). Percentage denotes the cells in each cell cycle phase from the total single population. Data from three independent experiments are summarized in bar graph format (D). Data are shown as mean ± SD (**P < 0.01 compared to control cells without thiocoraline treatment).

The cell cycle inhibitors, p21 and p27 have been shown to be crucial regulators of the cell cycle 24. Western blotting confirmed that levels of both p21 and p27 gradually increased in a dose-dependent fashion after thiocoraline treatment, indicating that the TT cells were undergoing cell cycle arrest (Fig. 3A). Additionally, cyclins B1 and D1 have been shown to be essential for successful completion of the cell cycle. Several studies reported that cyclin B1 was necessary for completion of the G2/M phase transition as well as M phase itself 25–28. However, cyclin B1 reduction has also been shown to cause cell cycle arrest at the G1 phase following celecoxib treatment in bladder cancer 29. Cyclin D1 has been shown to be essential for the execution of G1 phase 30–32. Analysis via Western blot clearly demonstrated that levels of both cyclin B1 and cyclin D1 were significantly decreased in response to thiocoraline treatment (Fig. 3B). These results implied that thiocoraline induced cell cycle arrest in TT cells. Having established that thiocoraline alters expression of cell cycle progression proteins, we proceeded to analyze the DNA content in all phases of the cell cycle. To investigate the specific cell cycle block, TT cells were treated with DMSO (control) or 5 and 10 nmol/L of thiocoraline for 48 h and then hypotonically lysed to stain the nuclear DNA with propidium iodide. Flow cytometry profiles of nuclear DNA content revealed a highly significant (*P* < 0.01) increase in the number of cells in G1 phase and decrease in the amount of cells in the S phase (Fig. 3C and D) for both concentrations of thiocoraline (5 and 10 nmol/L) compared to the control. These results suggested that thiocoraline suppressed TT cell growth by inducing specifically G1 arrest in cell cycle progression.

### Thiocoraline treatment increases expression of Notch isoforms

**Figure 4:**
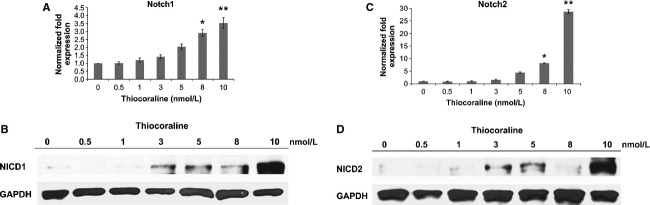
Thiocoraline induces expression of Notch isoforms. Thiocoraline increases NICD1 and NICD2 expression in a dose-dependent manner on transcript (A and C, respectively) and protein levels (B and D, respectively). NICD1 and NICD2 mRNA levels in thiocoraline-treated TT cells were assessed by real-time RT-PCR, and data were plotted relative to control cells without thiocoraline treatment. All values are expressed as mean ± SD (*P < 0.05 and **P < 0.01 compared with control). Western blot analysis was used to detect NICD1 and NICD2 protein expression levels. Equal loading was confirmed with GAPDH.

We have previously reported that Notch signaling is minimally active in TT cells and induction of Notch isoforms alters the malignant neuroendocrine phenotype 4,16. To test the potential role of thiocoraline as the inducer of Notch pathway in TT cells, we measured the expression of Notch1 and Notch2 isoforms on transcript and protein levels. Quantitative real-time PCR (RT-PCR) experiments showed that message levels of Notch1 and Notch2 were significantly (*P* < 0.05) and very significantly (*P* < 0.01) increased at 8 and 10 nmol/L dosages, respectively (Fig. 4A and C). In order to ensure the reliability of our results, the real-time PCR experiments were conducted two or more times and the results were averaged and represented as a normalized fold expression relative to the nontreated control. To determine whether the relative change in Notch1 and Notch2 transcripts correlated with protein expression, Western blotting was conducted for the Notch1 and Notch2 intracellular domains (NICD1 and NICD2). Notch intracellular domain (NICD) can be detected after successful drug induction increasing the amount of Notch receptors, followed by the interaction between the Notch receptor and its ligand followed by γ-secretase cleavage 20. Immunoblot analysis showed that thiocoraline treatment dramatically increased protein levels of NICD1 and NICD2 beginning with a dose of 3 nmol/L compared with untreated cells (Fig. 4B and D). Thus, these results indicated that the reinstitution of Notch expression could be a potentially therapeutic effect of thiocoraline treatment.

### The Notch pathway is functionally activated by thiocoraline treatment

**Figure 5:**
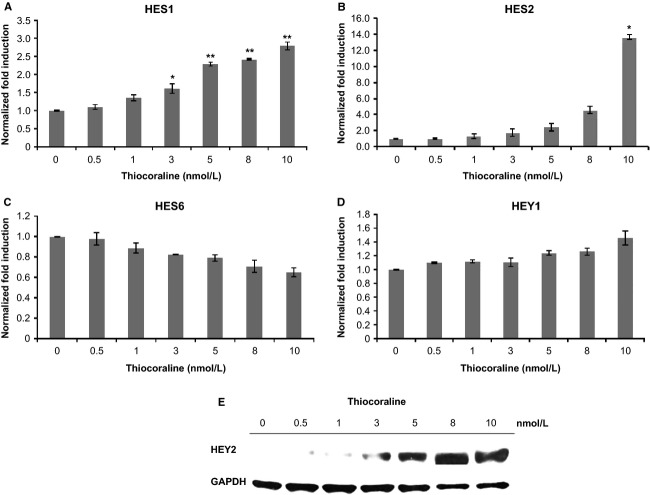
Thiocoraline functionally activates the Notch pathway. Real-time RT-PCR demonstrated activation of downstream targets of Notch pathway, HES1 (A), HES2 (B), HES6 (C), and HEY1 (D) in TT cells. While HES1 and HES2 transcript levels significantly increased with thiocoraline treatment comparing to the control (all values are expressed as mean ± SD with *P < 0.05 and **P < 0.01), decreasing values of HES6 and increasing values of HEY1 expression failed to reach statistical significance in comparison to the control. Western blot demonstrated an increase in HEY2 (E) at the protein level in TT cells.

Previous research has clearly demonstrated that the NICD fragment binds with CBF-1 and other proteins to modulate transcription of the HES and HEY families of genes 20,33. Specifically, activation of the Notch pathway resulted in decreased expression of HES6 as well as an increased expression of HES1 and HEY1 33,34. In order to assess if thiocoraline treatment functionally activated the Notch pathway, real-time PCR was conducted to assess the relative induction or reduction in mRNA levels of HES1, HES2, HES6, and HEY1. Analysis revealed that expression levels of HES1, HES2, and HEY1 increased (Fig. 5A, B, and D, respectively), and HES6 decreased (Fig. 5C) in a dose-dependent manner. HES1 mRNA expression was found to be significantly increased (*P* < 0.05) at 3 nmol/L treatment and very significantly increased (*P* < 0.01) at 5, 8, and 10 nmol/L and HES2 expression was significant at 10 nmol/L (*P* < 0.05). Moreover, HEY2 increased dose dependently with thiocoraline treatment at protein level. These results showed that the mRNA or protein levels of HES and HEY gene families were modified in a manner consistent with Notch induction as the dose of thiocoraline treatment was increased, suggesting that thiocoraline functionally activated the Notch pathway.

### Thiocoraline alters the expression of NET markers and inhibits NE-regulated secretory proteins

**Figure 6:**
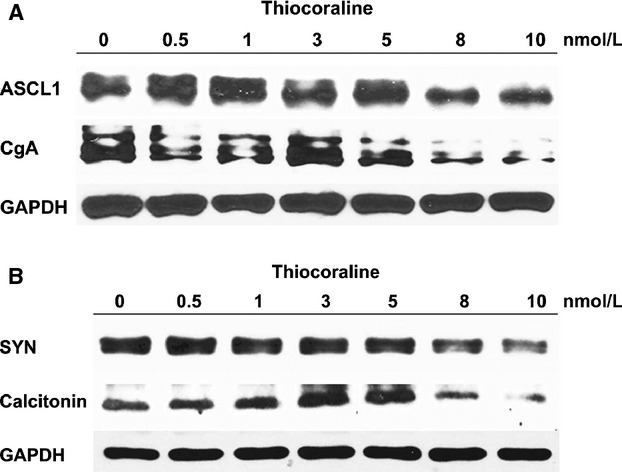
Thiocoraline alters the expression of NET markers and inhibits NE-regulated secretory proteins. Western blot analysis demonstrated a dose-dependent reduction in NET markers, ASCL1 and CgA (A) and suppression of NE-regulated secretory proteins, synaptophysin (SYP), and calcitonin comparing to the TT baseline (B). Equal loading was confirmed with GAPDH.

ASCL1 and CgA, NET markers, have been characteristically high in MTC, and their presence has been shown to be predictive of poor prognosis 4,9,10,35. Importantly, Notch expression was known to decrease levels of these tumor markers in TT cells 4. As a result, we used Western blotting to detect levels of NET markers after thiocoraline treatment and determined that ASCL1 and CgA levels decreased in a dose-dependent manner with the largest decrease found at 10 nmol/L for both proteins (Fig. 6A). Likewise, SYP and calcitonin, NE-regulated secretory proteins, have also been found to be highly expressed in MTC, and their high levels were correlated with a risk of metastatic progression 36. Western blotting showed that SYP and calcitonin levels were suppressed in a dose-dependent manner (Fig. 6B). Together, these results indicated that thiocoraline, in addition to inhibiting cancer cell proliferation, was capable of reducing NET markers correlated with poor prognosis and may relieve many of the symptoms associated with MTC.

## Discussion

Our results indicate that thiocoraline is capable of inducing a significant decrease in MTC-TT cell proliferation in vitro via cell cycle arrest and NET marker reduction likely due to functional activation of the Notch signaling pathway. The effects of thiocoraline on MTC have not been previously documented and our results suggest that thiocoraline has therapeutic potential. These preliminary results are especially important because there are limited treatments for MTC aside from surgical resection. Moreover, distal metastases frequently make surgical intervention impossible for MTC patients, giving new treatment options like thiocoraline of greater clinical significance.

There is a paucity of information as to the mechanism of action of thiocoraline in vitro and in vivo due to the fact that thiocoraline was first described very recently 37. It was originally discovered in 1997 and showed promise. The mechanism is mixed between inhibiting DNA polymerase and also inhibited RNA polymerase. The finding that there is an increase in protein expression of NICD1 and NICD2 and a change in expression of downstream Notch targets, as well as a reduction in ASCL1, CgA, SYP, and calcitonin levels strongly suggest that Notch signaling is functionally active and is at least one of the pathways through which the antiproliferative effects of thiocoraline are mediated. Previous research has shown that increased Notch signaling in the MTC-TT context decreases NET marker expression and the fact that thiocoraline treatment parallels these effects also provides strong evidence that thiocoraline is acting via the Notch pathway. The effects of Notch pathway activation have been shown to be context-dependent – acting in an oncogenic fashion in some types of cancer and functioning as a tumor suppressor in other tissues 38,39. Previous work has demonstrated that the Notch pathway is not active in MTC-TT cells and that institution of Notch signaling inhibits cell proliferation and reduces expression of NET markers 16,38.

The activation of Notch1 has been reported to cause cell cycle arrest in human tongue cancer cell lines. Transfected cells demonstrated a higher proportion of cells in G0–G1 phase, and a reduction in cells in the S phase 38. Our results follow this trend as thiocoraline-treated TT cells both activated Notch1 and Notch2 and demonstrated a decrease in an accumulation of cells in the S phase fractions. Previous studies have described the actions of thiocoraline on human colon adenocarcinoma as causing G1 arrest using flow cytometric analysis 13. Our findings support this observation in MTC-TT cells, as we found thiocoraline to increase TT cell population in G1 phase. In addition, we observed a decrease in expression of cyclin B1 and D1 proteins in response to thiocoraline treatment and increased expression of p21 and p27. Cumulatively, these results strongly suggest that mode of action of thiocoraline in TT cells through cell cycle arrest mediated by an induction of Notch signaling. Our results showed an increase in p21 and Notch1 protein expression with increasing thiocoraline treatment. Dotto 40 reported that increased Notch1 activity leads to increased p21 expression apparently mediated by the binding of CBF-1 to the p21 protein. In keratinocytes, experiments have shown increased p21 expression is essential for the suppressive growth effects of activated Notch1 41. In this study, we found thiocoraline-treated TT cells to exhibit reduced cell growth with an increase in p21, Notch1, and Notch2 protein expression. In addition, Lefort and Dotto 41 also concluded that the ability of keratinocyte stem cells to differentiate is controlled by the overlap of Notch1 activation and p21 expression. These results reflect the first identification of the signaling pathway, Notch, by which thiocoraline exerts its effects.

Importantly, thiocoraline exhibits its effects on TT cell phenotype at concentrations below 10 nmol/L – a relatively small concentration that is likely achievable in vivo. Faircloth et al. 42 tested thiocoraline treatment on three separate cancer cell lines including human colon, human NSCLC (non–small cell lung cancer cell line), and melanoma cell lines and determined IC_50_ values of 2.5 nmol/L. In experiments by Erba et al., thiocoraline was tested on two different human colon adenocarcinoma cell lines with IC_50_ values of 15 (SW620 cells) and 500 nmol/L (LoVo) 13. It is clear that cancer cell lines show a wide range of sensitivities to thiocoraline, but generally, the low IC_50_ values are realistic to test in vivo and provide encouraging evidence that thiocoraline may be effective as a therapeutic in the clinical setting. Additionally, because thiocoraline treatment causes a reduction in NET markers as well as cell proliferation, it is reasonable to predict that patients would experience a decrease in cancer progression and painful symptoms associated with NET marker overproduction.

Thiocoraline is a relatively hydrophobic compound and there have been limited studies assessing its in vivo effects. Natural analogs of thiocoraline which have been made more hydrophilic had decreased potency 12. It is known that the half-life of thiocoraline in human plasma is close to 4 h as compared to that of PBS solution of about 25 h 15,37. In addition thiocoraline, contains many potential sites of degradation, with a P450 isozyme involved in thiocoraline metabolism 15. Further research will need to address the hydrophobicity of the compound to ensure that sufficient dosages are delivered to the cancer site to exert Notch activation and subsequent antiproliferative effects.

In conclusion, we show for the first time that thiocoraline treatment in MTC-TT cells has an antiproliferative effect caused by cell cycle arrest and induces a decrease in NET production. Both of these effects are consistent with the observed induction of Notch signaling. Moreover, thiocoraline is effective at low nanomolar concentrations in vitro. As a result, future research should focus on continued preclinical testing of thiocoraline in both in vitro and in vivo models in the hope of providing palliative or curative treatment to patients with MTC.

## Conflict of Interest

None declared.
